# Healthy aging profile in octogenarians in Brazil**^1^**

**DOI:** 10.1590/1518-8345.0694.2724

**Published:** 2016-08-29

**Authors:** Ana Cristina Viana Campos, Efigênia Ferreira e Ferreira, Andréa Maria Duarte Vargas, Lúcia Hisako Takase Gonçalves

**Affiliations:** 2 PhD, Professor Adjunto, Faculdade de Ciências da Saúde e Biológicas, Universidade Federal do Sul e Sudeste do Pará, Marabá, PA, Brazil.; 3 PhD, Adjunct Professor, Faculdade de Odontologia, Universidade Federal de Minas Gerais, Belo Horizonte, MG, Brazil.; 4 PhD, Associate Professor, Faculdade de Odontologia, Universidade Federal de Minas Gerais, Belo Horizonte, MG, Brazil.; 5 PhD, Researcher Volunteer, Universidade Federal do Pará, Belém, PA, Brazil.

**Keywords:** Aging, Aged, 80 and Over, Health Profile, Epidemiologic Factors, Epidemiology

## Abstract

**Objective:**

to identify the healthy aging profile in octogenarians in Brazil.

**Method:**

this population-based epidemiological study was conducted using household interviews of 335 octogenarians in a Brazilian municipality. The decision-tree model was used to assess the healthy aging profile in relation to the socioeconomic characteristics evaluated at baseline. All of the tests used a p-value < 0.05.

**Results:**

the majority of the 335 participating older adults were women (62.1%), were aged between 80 and 84 years (50.4%), were widowed (53.4%), were illiterate (59.1%), had a monthly income of less than one minimum wage (59.1%), were retired (85.7%), lived with their spouse (63.8%), did not have a caregiver (60.3%), had two or more children (82.7%), and had two or more grandchildren (78.8%). The results indicate three age groups with a healthier aging profile: older adults aged 80 to 84 years (55.6%), older adults aged 85 years and older who are married (64.9%), and older adults aged 85 and older who do not have a partner or a caregiver (54.2%).

**Conclusion:**

the healthy aging profile of octogenarians can be explained by age group, marital status, and the presence of a caregiver.

## Introduction

The aging of the world population is occurring heterogeneously. In 1999, 10% of the population was aged 60 years and older, ranging from 19% in developed countries to 5% in developing countries. The United Nations estimates that this percentage will double by 2050^1^. 

The last Brazilian census confirms that this process is occurring faster in Brazil than it occurred in Europe at the beginning of the demographic transition. In 2000, the population aged over 60 years corresponded to 8.6% of the total population, whereas this percentage increased to 10.8% in the 2010 census. Adults older than 80 years constitute the age group with the highest percentage of growth in recent years, representing 14.3% of older adults in Brazil and 1.5% of the total population of Brazil in 2010^2^.

Camarano^3^ ^)^ reported that the elderly population is also aging, i.e., the proportion of those aged ≥ 80 years is also increasing, thereby changing the age composition of the group. The analysis of the different trajectories of life of older people reveals that they fall into different social and economic statuses in Brazil. 

However, few studies in this area have investigated the lifestyle and health status of older adults of this new age stratum. This context challenges governments and society to pursue actions and health promotion policies considering a broader perspective of health and well-being in old age^4^. The concept of healthy aging can help explain these demographic and epidemiological changes. 

From a broader perspective, healthy aging is a continuous process of learning and personal development aimed at achieving autonomy and independence for elderly people^4^. This process also involves the balance of the interactions between the various dimensions of life of this age group: physical and mental health, independence and autonomy in activities of daily living, social involvement and support, family interaction and support, and economic independence^5^.

A survey conducted in a subsample of the Healthy Aging Processes (Processos do Envelhecimento Saudável-PENSA) study sought to investigate how older people perceive the multiple dimensions of the concept of healthy aging. Regarding the factors considered necessary to achieve healthy aging, the categories most frequently mentioned by older people were physical health (53%), social health (46%), emotional health (37%), concern about nutrition and exercise (36%), and the prevention of risk factors (19%)^6^.

In practice, the lack of parameters related to functional, cognitive, physical, and social aspects, as well as the lack of physiological parameters, has limited the identification of healthy older adults^7^, particularly among octogenarians.

Therefore, the aim of this study was to assess the healthy aging profile of octogenarians in Brazil.

## Methods

The Aging, Gender, and Quality of Life (AGEQOL) study is a population-based cohort study conducted in Sete Lagoas, state of Minas Gerais, Brazil, in 2012, with a sample representative of the older population of the municipality^8^.

Sampling was performed in two stages: in the first stage, census sectors were selected; in the second stage, households were selected, and their number was proportional to the size of each sector. In each household, all residents aged 60 years and older were interviewed, regardless of their marital status or degree of kinship. Data were collected by trained researchers via interviews with the older adults in their homes. 

This study followed the ethical guidelines for research with human beings and was approved by the Research Ethics Committee of the Federal University of Minas Gerais (Universidade Federal de Minas Gerais - UFMG) under the Certificate of Presentation for Ethical Appreciation (Certificado de Apresentação para Apreciação Ética-CAAE) No. 0413.0.203.000-11. All of the study participants signed an informed consent form^8^.

**Figure 1:**
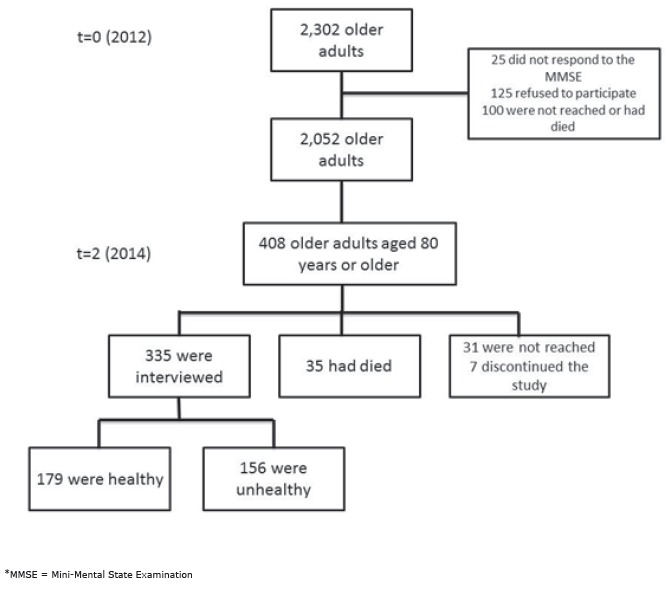
Flow diagram of the AGEQOL study. Sete Lagoas, state of Minas Gerais, Brazil

Of the selected participants (N = 2,302), 250 were lost to the first follow-up in 2012. In 2014, a new data collection was performed in the homes of individuals aged 80 years and older. The data presented herein were obtained from 408 octogenarians, of whom 335 (81.5%) were interviewed, 35 (8.5%) had died, 31 (7.6%) were not reached after three visits, and 7 (1.7%) discontinued the study (Figure 1). 

The outcome variable of the study sample was healthy aging. This variable was evaluated using a structured questionnaire applied in the homes of the participants between January and March 2014. This variable was constructed with adaptations from the study conducted by Carrasco^7^. The protocol was developed by a team of geriatric medicine experts to identify healthy individuals from the community considering the principles of healthy aging together with a low comorbidity burden. In this study, the following criteria were used to identify a healthy older adult: positive self-perceived health (good and very good); functionally independent for all daily and instrumental activities; not suffering from cognitive impairment; capable of walking at least three blocks without assistance; no acute or chronic diseases; taking fewer than three medications; not smoking (never having smoked or having smoked but quit); and rarely consuming alcohol (none or less than one day per week).

Self-perceived health was assessed using a Likert scale for the responses (very poor, poor, fair, good, and very good). Mobility was assessed using a single question: "Can you walk three blocks without assistance?", with a dichotomous response (yes or no). The use of medications was evaluated by the number of drugs taken at the time of the study. A dichotomous question was used to assess the presence or absence of chronic or acute diseases known at the time of the study. The frequency of alcohol intake was evaluated for the three months before the beginning of the study using the following question: "On average, how many days a week do you consume alcoholic beverages such as beer, wine, liquor, and sugarcane rum?" The response categories were none, less than one day per week, one day per week, two to three days per week, four to six days per week, and every day. With regard to smoking, the participants responded whether they 1) never smoked, 2) smoked but quit, 3) smoked occasionally, i.e., less than one cigarette per day, or 4) currently smoked, i.e., at least one cigarette per day^7^.

To assess cognitive status, the Mini-Mental State Examination (MMSE) validated in Brazil^9^ was used with a cutoff value of 21/22 points^10^. A score of 21 points or less indicated cognitive impairment. Functional limitation was investigated via the assessment of six basic activities of daily living (ADLs) (bathing, dressing, toileting, eating, lying down and getting out of bed or a chair, and urinary and fecal incontinence) and eight instrumental activities of daily living (IADLs) (using the phone, using transportation, shopping, cooking, cleaning the house, doing laundry, taking care of finances, and taking medications). The classification of "without limitations" was applied to individuals with complete independence in performing ADLs and IADLs, separately.

The socioeconomic characteristics evaluated at baseline were age at the time of the study (80-84 years, ≥85 years), gender (male, female), marital status (married, separated, single, widowed), self-reported ethnicity (Caucasian, Black/Mixed, East Asian/Indigenous), level of education (literate, illiterate), monthly income (no income, ≤ 1 minimum wage, > 1 minimum wage), retired (yes, no), living arrangements (living with spouse, mixed arrangement, living alone), number of children (0, 1, ≥ 2), number of grandchildren (0, 1, ≥ 2), and the presence of a caregiver (yes, no). 

Initially, the data were analyzed descriptively. The associations between the variables of interest were evaluated using the Chi-square test at a level of significance of 5%.

The decision-tree model was used to analyze the healthy aging profile relative to the other predictors. This method consists of decision rules used to successively subdivide the dataset to make it increasingly homogeneous for the outcome variable. The decision tree is presented in the form of a graph and starts with a root node that includes all the characteristics of the study sample. The nodes produced in sequence represent subdivisions of the data in groups that are *increasingly homogeneous* ^11^.

The method used was the Chi-squared Automatic Interaction Detector (CHAID) algorithm, which can detect and record the non-linear effects on the response variable and the interactions between the predictors. The interpretation of the tree focuses primarily on analyzing the group with the largest number of individuals formed by the last node of the tree, which represents the final result of the model^12^.

The model was fitted by successive binary divisions (nodes) of the datasets. The stop criterion was a p-value < 0.05 using the Chi-square statistic and Bonferroni correction. The final fit was assessed by estimating the overall risk, which compared the difference between the expected and observed values in the model, indicating to what extent the decision tree predicted the results correctly. All of the analyses were performed using SPSS software version 19.0 (SPSS Inc., Chicago, United States).

## Results

The age of the study sample in 2014 ranged between 80 and 108 years, with a mean age of 85.2 ± 4.6 years (85.3 ± 4.8 for women and 85.1 ± 4.1 for men). 

Of the 335 participating older adults, the majority were women (62.1%), were between the ages of 80 and 84 (50.4%), were widowed (53.4%), were illiterate (59.1%), had a monthly income of less than one minimum wage (59.1%), were retired (85.7%), lived with their spouse (63.8%), did not have a caregiver (60.3%), had two or more children ( 82.7%), and had two or more grandchildren (78.8%) (Table 1).

Table 1 shows the association between gender and the following variables: ethnicity (p = 0.035), marital status (p < 0.001), being retired (p < 0.001), and living alone (p = 0.041). This association indicated a profile of women who were widowed, retired, and lived in mixed arrangements or alone.

**Table 1:** Socioeconomic and demographic characteristics of the study sample. Sete Lagoas, state of Minas Gerais, Brazil, 2014

Variables		Total (N = 335)		Men (N = 127)		Women (N = 208)		p-value*
		N	%	N	%	N	%	
Age								0.988
	80-84 years	169	50.4	64	50.4	105	50.5	
	≥ 85 years	166	49.6	63	49.6	103	49.5	
Self-reported ethnicity								0.035
	Caucasian	139	41.5	61	48.0	78	37.5	
	Black/Mixed	44	13.1	20	15.7	26	12.5	
	East Asian/Indigenous	149	44.5	44	34.6	103	49.5	
	Did not respond	3	0.9	02	1.6	1	0.5	
Marital status								<0.001
	Married	120	35.8	81	63.8	39	18.8	
	Separated	13	3.9	7	5.5	6	2.9	
	Widowed	179	53.4	36	28.3	143	68.8	
	Single	23	6.9	3	2.4	20	9.6	
Level of education								0.989
	Literate	137	40.9	52	49.9	85	49.9	
	Illiterate	198	59.1	75	59.1	123	59.1	
Monthly income								0.671
	No income	32	9.6	10	7.9	22	10.6	
	≤ 1 minimum wage^†^	198	59.1	75	59.1	123	59.1	
	> 1 minimum wage^†^	105	31.3	42	33.1	63	30.3	
Retired								<0.001
	Yes	287	85.7	122	96.1	165	79.3	
	No	48	14.3	5	3.9	43	20.7	
Living arrangement								<0.001
	Living with spouse	81	63.8	37	17.8	118	35.2	
	Mixed arrangement	30	23.6	126	60.6	156	46.6	
	Living alone	12	9.4	35	16.8	47	14.0	
	Did not respond	4	3.1	10	4.8	14	4.2	
Number of children								0.367
	0	36	10.7	10	7.9	26	12.5	
	1	13	3.9	6	4.7	7	3.4	
	≥ 2	277	82.7	107	84.3	170	81.7	
	Did not respond	9	2.7	4	3.1	5	2.4	
Number of grandchildren								0.787
	0	44	13.1	15	11.8	29	13.9	
	1	9	2.7	3	2.4	6	2.9	
	≥ 2	264	78.8	103	81.1	161	77.4	
	Did not respond	18	5.4	6	4.7	12	5.8	
Caregiver								0.431
	Yes	133	39.7	47	37.0	86	41.3	
	No	202	60.3	80	63.0	122	58.7	

*Chi-square test with correction using Fisher's exact test

†Brazilian minimum wage corresponds to BRL 622.00 (~USD 300)

The prevalence of cognitive impairment in the study sample was 27.2%, and functional limitation was higher for IADLs (55.5%) than for ADLs (24.5%). Although most of the older adults evaluated reported having no chronic or acute diseases (57.1%), only 37.4% reported not taking any medications (Table 2). 

**Table 2:** Health status and lifestyle of the study sample. Sete Lagoas, state of Minas Gerais, Brazil, 2014

Variables		Total (N = 335)		Men (N = 127)		Women (N = 208)		p-value*
		N	%	N	%	N	%	
Self-perceived health								0.798
	Very poor	18	5.4	5	3.9	13	630	
	Poor	37	11.0	14	11.0	23	11.1	
	Fair	122	36.4	45	35.4	77	37.0	
	Good	142	42.4	58	45.7	84	40.4	
	Very good	16	4.8	5	3.9	11	5.3	
Cognitive impairment								0.311
	Yes	91	27.2	30	23.6	61	29.3	
	No	244	72.8	97	76.4	147	70.7	
Limitations in ADLs ††								0.063
	Yes	82	24.5	24	18.9	58	27.9	
	No	253	75.5	103	81.1	150	72.1	
Limitations in IADLs‡								0.426
	Yes	186	55.5	67	52.8	119	57.2	
	No	149	44.5	60	47.2	89	42.8	
Could walk three blocks without assistance								0.621
	Yes	142	42.4	56	44.1	86	41.3	
	No	193.0	57.6	71	55.9	122	58.7	
Frequency of alcohol intake								0.007
	Never	165	49.2	60	47.2	105	50.5	
	Less than one day per week	150	44.8	53	41.7	97	46.2	
	One day per week	6	1.8	4	3.1	02	1.0	
	Two to three days per week	6	1.8	4	3.1	02	1.0	
	Four to six days per week	2	0.6	0	0	2	1.0	
	Every day	6	1.8	6	4.7	0	0	
Smoking status								<0.001
	Never smoked	183	56.3	56	44.8	127	63.5	
	Smoked but quit	90	27.7	52	41.6	38	19.0	
	Smoked occasionally (less than one cigarette per day)	29	8.9	10	8.0	19	9.5	
	Current smoker (at least one cigarette per day)	23	7.1	7	5.6	16	8.0	
Presence of chronic or acute diseases								0.225
	Yes	103	30.7	34	26.8	69	33.2	
	No	232	69.3	93	73.2	139	66.8	
Number of medications taken								0.135
	0	123	36.7	54	42.5	69	33.2	
	1-3	120	35.8	46	36.2	74	35.6	
	≥ 4	86	25.7	26	20.5	60	28.8	
	Did not respond	6	1.8	1	0.8	5	2.4	

*Chi-square test with correction using Fisher's exact test

†ADLs = activities of daily living

‡IADLs = instrumental activities of daily living

With regard to self-perceived health, 47.2% rated their health as good or very good, 36.4% rated it as fair, and 16.4% rated is as poor or very poor. Only 42.4% of octogenarians could walk three blocks without assistance. Most of the older adults evaluated did not drink alcoholic beverages or did so less than one day per week (94.0%), and 56.3% had never smoked (Table 2).

The analysis between the genders (Table 2) indicated that most women did not consume alcohol (50.5%) whereas 10.9% of men consumed alcohol at varying frequencies (p = 0.007). With regard to smoking, 63.5% of the women and 44.8% of the men had never smoked (p < 0.001).

Healthy aging was significantly associated with being married (p = 0.015) and not having a caregiver (p = 0.014) (Table 3).

**Table 3:** Healthy aging in the study group according to socioeconomic and health statuses. Sete Lagoas, state of Minas Gerais, Brazil, 2014

Variables		Healthy aging		Unhealthy aging		p-value*
		N	%	N	%	
Age (N = 335)						0.444
	80-84 years	94	52.5	75	48.1	
	≥ 85 years	85	47.5	81	51.9	
Gender (N = 335)						
	Men	75	41.9	52	33.3	0.115
	Women	104	58.1	104	66.7	
Marital status (N = 335)						
	Married	76	42.5	44	28.2	0.015
	Separated	8	4.5	5	3.2	
	Widowed	81	45.3	98	62.8	
	Single	14	7.8	9	5.8	
Self-reported ethnicity (N = 332)						
	Caucasian	76	42.7	63	40.9	0.944
	Black/mixed	23	12.9	21	13.6	
	East Asian/Indigenous	79	44.4	70	45.5	
Level of education (N = 335)						
	Literate	103	57.5	95	60.9	0.578
	Illiterate	76	42.5	61	39.1	
Monthly income (N = 335)						
	No income	15	8.4	17	10.9	0.668
	≤ 1 minimum wage^†^	109	60.9	89	57.1	
	> 1 minimum wage^†^	55	30.7	50	32.1	
Retired (n = 335)						
	Yes	157	87.7	130	83.3	0.276
	No	22	12.3	26	16.7	
Caregiver (N = 335)						
	Yes	60	33.5	73	46.8	0.014
	No	119	66.5	83	53.2	
Living arrangement (N = 321)						
	Living with spouse	73	42.4	45	30.2	0.076
	Mixed arrangement	76	44.2	80	53.7	
	Living alone	23	13.4	24	16.1	
Number of children (N = 326)						
	0	24	13.7	12	7.9	0.251
	1	7	4.0	6	4.0	
	≥ 2	144	82.3	133	88.1	
Number of grandchildren (N = 317)						
	0	28	16.4	16	11.0	0.260
	1	6	3.5	3	2.1	
	≥ 2	137	80.1	127	87.0	

*Chi-square test with correction using Fisher's exact test

†Brazilian minimum wage corresponds to BRL 622.00 (~USD 300)

**Figure 2:**
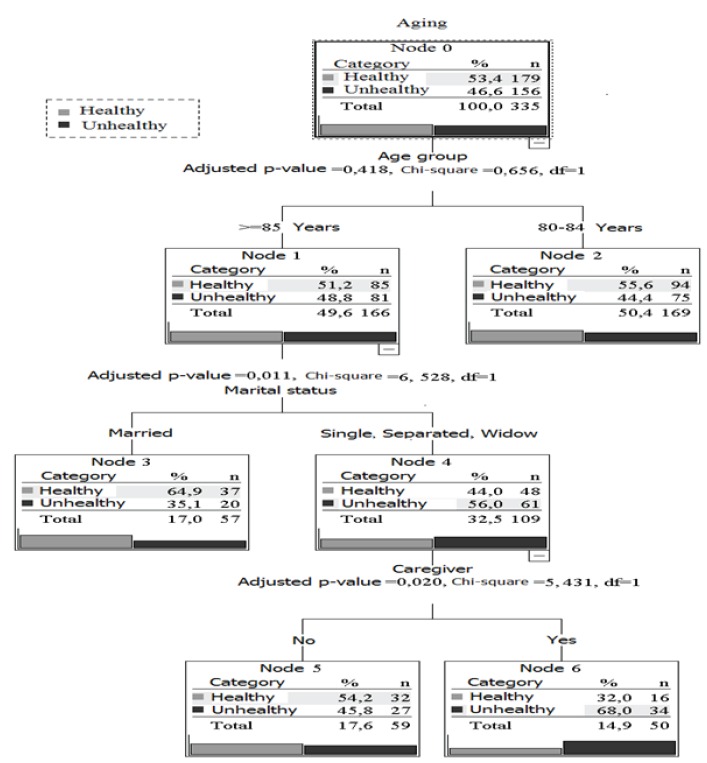
Multivariate analysis using a decision tree (CHAID) for healthy aging in octogenarians, adjusted for socioeconomic factors. Sete Lagoas, state of Minas Gerais, Brazil, 2014

The tree model was built with eight nodes, the estimated average risk was 0.412 (± 0.027), and the total correct classification was 60.0%. The healthy aging profile of octogenarians could be explained by age, marital status, and the presence or absence of a caregiver. The first node gave rise to two distinct branches, which described healthy aging according to the age groups: individuals aged 80-84 years (N = 169; 50.4%) and individuals aged ≥ 85 years (N = 166; 49.6%). In general, healthy aging prevailed among subjects in the 80-84 year age group (55.6%), among those who were married (64.9%), and among those without partners or caregivers (54.2%). Unhealthy aging prevailed in 34 (68.0%) older adults without partners and with caregivers (Figure 2).

## Discussion

In general, the profile of the population studied was similar to that of previous studies conducted in Brazil^4^ ^,^ ^13^ and other countries^14^ ^-^ ^16^. The majority of the participants were women, widows, had a low education level, were retired, had an income of less than one minimum wage, and were living with family members or caregivers. 

In the last Brazilian population census, the illiteracy rate among older people was 26.2%^17^, and this rate is similar to that found in the AGEQOL study (28.2%)^8^. These values were even higher (59.1%) in the ≥ 80 year age group, as reported in other studies^4^ ^,^ ^13^. These differences in the literacy level reflect the social inequalities in the early twentieth century, a time when these older adults should have been in school, but education was not available to the poor and women.

Among the octogenarians of this study, there were statistical differences between the genders for marital status and the type of living arrangement. Most of the men were married (63.8%) whereas 143 (68.8%) women were widows, and 77.1% of the latter lived alone. This result is similar to that reported in other Latin American cities evaluated in the Health, Welfare, and Aging (Saúde, Bem-estar e Envelhecimento-SABE) study^18^ and in other Brazilian studies involving octogenarians^19^ ^-^ ^20^. 

The condition of living alone was a cause for concern and was more frequent among women, possibly because of the greater likelihood of remarrying observed among men^18^, which does not occur among women. These data demonstrate the importance of adjusting to a new family arrangement in this age group^20^.

In addition, substantial differences were observed between the genders for smoking and alcohol consumption. Although most of the older people evaluated in this study had never smoked (56.3%), 41.6% of the men were former smokers. Other inferences could not be made at this time because the data on the period that this group had smoked and when they had quit were not assessed.

A study involving 832 individuals aged 60 and older living in Porto Alegre, state of Rio Grande do Sul, Brazil, revealed a higher prevalence of smoking and alcohol consumption among men (11.7 and 20.8%, respectively) compared to the prevalence in women (0.7 and 13.0%, respectively)^21^. The studies on smoking and alcohol consumption and their consequences among older people suggest correlations with gender, ethnicity, and socioeconomic status^22^.

In this study, the concept of healthy aging included an adequate perception of health, independence to perform ADLs, absence of cognitive impairment, healthy lifestyle and habits (not smoking or drinking alcohol and taking few medications), adequate mobility, and lack of morbidities.

Previous studies have shown that healthy behaviors, including not smoking, management of weight and blood pressure, and regular exercise, are associated with healthy aging and improved quality of life in older people^23^ ^-^ ^25^. For this reason, it is necessary to invest in individual and public health interventions to guide these subjects and to develop new strategies to ensure a longer and healthier life for future generations^26^.

Notably, we expected to find gender differences for healthy aging among octogenarians in the tree model, based on the literature on aging^8^ ^,^ ^27^ ^-^ ^28^.

However, the results indicate the presence of three groups with a healthier aging profile: older people aged 80-84 years (55.6%), those aged 85 years or older and married (64.9%), and those aged 85 or older who do not have a partner or a caregiver (54.2%).

The second group comprised individuals aged > 85 years who were married and healthy, suggesting that marriage can be a positive factor for healthy aging among octogenarians. Our results suggest that the relationship between husband and wife should be assessed and monitored by health professionals as a strategy to prevent functional dependence in octogenarians.

Studies with married older adults have been conducted to identify the determinants of happiness, health, and well-being in old age. The study by Waldinger and Schulz^29^ revealed that in both genders, marital satisfaction was strongly associated with a more positive daily link between the time spent with their partner and the level of happiness. Another study conducted in China with octogenarians revealed a high prevalence (62.4%) of married older adults with a better psychological well-being^16^.

The stratum of unhealthy subjects older than 85 years was associated with being single, widowed, or divorced (p = 0.011) and requiring the assistance of a caregiver (p = 0.020). These results indicate that this age group comprised older adults with more morbidities and functional limitations, who required more care, and did not have a partner to share their life with and help in daily activities.

The caregiver is often a close relative and someone of the female gender (spouses, daughters, and granddaughters) who resides in the home of the older adults and becomes responsible for all aspects of their life^30^. Intervention actions in this age group are unique and should enable family members and formal caregivers to better address the difficulties related to functional, physical, cognitive, and psychological limitations of older individuals.

Living alone is a risk factor for depressive symptoms and decline in psychological well-being among older adults^16^. Therefore, older people with a stable and reliable family support system can build strong family relationships and better overcome possible losses during the aging process^21^.

The limitations of this study include the lack of genetic information and the lack of corroboration with baseline data. The lack of previous data on the variables that compose the concept of healthy aging in this study prevented the separate calculation of survival for the healthy and unhealthy groups. The healthy aging profile was investigated using subjective self-reported information, which could lead to recall bias. 

Nonetheless, this is one of the few studies that has evaluated octogenarians using baseline data from a random sample with a high response rate to make inferences using complex statistical tools such as the decision tree.

The incorporation of other variables and the geoprocessing of data can help broaden the discussion and establish a temporal relationship between healthy aging and marital status among octogenarians in Brazil. 

Considering that marital status was an important determinant of the healthy aging profile in this sample, other data should be evaluated, including the subtypes of family arrangements (with two or three generations), the reasons for living at home, and whether these older adults were the head of the household^20^.

Further research should better assess the different requirements of and formulate public policies for this age group, considering the heterogeneity of this population regarding age and socioeconomic status^3^.

## Conclusion

Healthier older adults had a positive self-perceived health; the absence of functional impairment, cognitive impairment, and other morbidities; adequate mobility; and healthy habits. In the final model, this profile was determined by age, marital status, and the need for a caregiver. 

The concept of healthy aging adopted here can be considered a reliable and practical model in epidemiological studies on aging and for the reception and screening assessments of primary health care services.

Therefore, the results of this study should guide and improve future research on octogenarians and should also be used to establish new proposals for the development of policies on healthy aging targeting this age group, with a focus on marital status and family relations as a care unit. 

The care of life and health in the aging process is required, particularly in nursing because the nurses who work in primary care should focus on continued care throughout the life of the aging adult. The results of this epidemiological study indicate that the nurse should continue to invest in the promotion of healthy aging, seeking to extend the health and welfare of octogenarians beyond the first five years of the eighties. In addition, the nurse should pay attention to the continued care of older adults whose health has been impaired by morbidities or the natural weakening process because of increasing age and should provide strategies to improve the relationship between older adults and family caregivers.

## References

[1] Tinker A (2002). The social implications of an ageing population. Mech Ageing Dev.

[2] Instituto Brasileiro de Geografia e Estatística (2013). Atlas do Censo Demográfico 2010.

[3] Camarano AM, Kanso S, Mello JL, Camarano AM (2004). Como vive o idoso brasileiro?. Os Novos Idosos Brasileiros: Muito Além dos 60?.

[4] Inouye K, Pedrazzani ES (2007). Instruction, social economic status and evaluation of some dimensions
of octogenarians' quality of life Rev. Latino-Am. Enfermagem.

[5] Santos FH, Andrade VM, Bueno OFA (2009). Envelhecimento um processo multifatorial. Psicol Estud.

[6] Cupertino APFB, Rosa FHM, Ribeiro PCC (2007). Definição de Envelhecimento Saudável na Perspectiva de Indivíduos
Idosos. Psicol Reflex Crit.

[7] Carrasco M, Martínez G, Foradori A, Hoyl T, Valenzuela E, Quiroga T (2010). Identificación y caracterización del adulto mayor
saludable. Rev Med Chile.

[8] Campos ACV, Ferreira EF, Vargas AMD, Albala C (2014). Aging, Gender and Quality of Life (AGEQOL) study factors associated
with good quality of life in older Brazilian community-dwelling
adults. Health Qual Life Outcomes.

[9] Brucki SMD, Nitrini R, Caramelli P, Bertolucci PHF, Okamoto IH (2003). Sugestões para o uso do mini-exame do estado mental no
Brasil. Arq. Neuropsiquiatr.

[10] Quiroga P, Albala C, Klaasen G (2004). Validación de un test de tamizaje para el diagnóstico de demencia
asociada a edad, en Chile. Rev Med Chil.

[11] Hair JF, Black WC, Babin JB, Anderson RE, Tatham RL (2009). Análise Multivariada de Dados.

[12] Ritschard G. (2010). CHAID and Earlier Supervised Tree Methods. Cahiersdudépartement
d'économétrie.

[13] Tavares DMS, Ferreira PCS, Dias FA, Oliveira PB. (2014). Caracterização e distribuição espacial de homens
octogenários. Rev Enferm UERJ.

[14] Montez JK, Berkman LF (2014). Trends in the educational gradient of mortality among US adults aged
45 to 84 years bringing regional context into the explanation. Am J Public Health.

[15] Fleming J, Zhao E, O'Connor DW, Pollitt PA, Brayne1 C (2007). Cohort Profile: The Cambridge City over-75s Cohort
(CC75C). Int J Epidemiol.

[16] Wang J, Chen T, Han B (2014). Does co-residence with adult children associate with better
psychological well-being among the oldest old in China. Aging Ment Health.

[17] Ministério da Saúde (BR). Departamento de Informática do SUS
(DATASUS) (2011). Taxa de analfabetismo - B.1.

[18] Duarte YAO, Lebrão ML, Lima FD (2005). Contribuição dos arranjos domiciliares para o suprimento de demandas
assistenciais dos idosos com comprometimento funcional em São Paulo,
Brasil. Pan Am J Public Health.

[19] Pavarini SCI, Barha EJ, Mendiondo de MSZ, Filizola CLA, Petrilli JF, Santos dos AA (2009). Family and social vulnerability a study with octogenarians. Rev.
Latino-Am. Enfermagem.

[20] Pedrazzi EC, Motta TTD, Vendrúscolo TRP, Fabrício-Wehbe SCC, Cruz IR, Rodrigues RAP (2010). Household arrangements of the elder elderly Rev.
Latino-Am. Enfermagem.

[21] Senger AEV, Ely LS, Gandolfi T, Schneider RH, Gomes I, De Carli GA (2011). Alcoolismo e tabagismo em idosos relação com ingestão alimentar e
aspectos socioeconômicos. Rev Bras Geriatr Gerontol.

[22] Cummings SM, Cooper RL, Johnson C (2013). Alcohol misuse among older adult public housing
residents. J Gerontol Soc Work.

[23] Rizzuto D, Fratiglioni L (2014). Lifestyle factors related to mortality and survival a
mini-review. Gerontology.

[24] Peralta-Catipon T, Hwang JE (2011). Personal factors predictive of health-related lifestyles of
community-dwelling older adults. Am J Occup Ther.

[25] Davies N (2011). Promoting healthy ageing the importance of lifestyle. Nurs Stand.

[26] Peel NM, McClure RJ, Bartlett HP (2005). Behavioral determinants of healthy aging. Am J Prev Med.

[27] Belon AP, Lima MG, Barros MBA (2014). Gender differences in healthy life expectancy among Brazilian
elderly. Health Qual Life Outcomes.

[28] Boerner K, Jopp DS, Carr D, Sosinsky L, Kim SK (2014). "His" and "her" marriage? The role of positive and negative marital
characteristics in global marital satisfaction among older adults. J Gerontol B Psychol Sci Soc Sci.

[29] Waldinger RJ, Schulz MS (2010). What's love got to do with it Social functioning, perceived health,
and daily happiness in married octogenarians. Psychol Aging.

[30] Gonçalves LHT, Costa MAM, Martins MM, Nassar SM, Zunino R (2011). The family dynamics of elder elderly in the context of Porto, Portugal
Rev. Latino-Am. Enfermagem.

